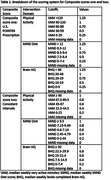# Development of a Multidomain Composite Intervention Engagement Score in the U.S. POINTER Trial

**DOI:** 10.1002/alz.090791

**Published:** 2025-01-09

**Authors:** Mina Ghadimi Nouran, Jeffrey A. Katula, Jennifer Ventrelle, Sarah Graef, Katelyn R Garcia, Laura Lovato, Sharon Wilmoth, Nancy Woolard, Xiaoyan Leng, Ismael Luis Calandri, Heather M Snyder, Laura D Baker

**Affiliations:** ^1^ Wake Forest University School of Medicine, Winston Salem, NC USA; ^2^ Wake Forest University School of Medicine, Winston‐Salem, NC USA; ^3^ Wake Forest University, Winston Salem, NC USA; ^4^ Rush University Medical Center, Chicago, IL USA; ^5^ Rush University, Chicago, IL USA; ^6^ Wake Forest School of Medicine, Winston‐Salem, NC USA; ^7^ Fleni, Buenos Aires, Buenos Aires Argentina; ^8^ Alzheimer Center Amsterdam, Amsterdam UMC, Amsterdam Netherlands; ^9^ Amsterdam Neuroscience, Neurodegeneration, Amsterdam Netherlands; ^10^ Fleni, Buenos Aires Argentina; ^11^ Amsterdam Neuroscience, Brain Imaging, Amsterdam Netherlands; ^12^ Alzheimer’s Association, Chicago, IL USA; ^13^ Wake Forest University, Winston‐Salem, NC USA; ^14^ Wake Forest University Health Sciences, Winston Salem, NC USA

## Abstract

**Background:**

Multidomain lifestyle interventions have shown promise to slow cognitive decline and possibly prevent dementia. However, challenges arise in analyzing and interpreting treatment response when participants vary in their adherence to intervention components. The U.S. POINTER trial, a phase 3, multicenter, randomized 2‐year clinical trial, is investigating the impact of lifestyle interventions on cognition in older adults at risk of cognitive decline.

**Methods:**

Four composite scores are proposed to assess engagement in the POINTER multidomain intervention.

**Results:**

Composite score one was based on the **U.S. POINTER Prescription** adherence goals for three intervention domains (physical activity, diet, brain training). For this composite score, values range from 0 to 1.25, where “1” signifies the adherence goal was met, and “1.25” indicates the goal was exceeded (Table 1). The composite score is the sum of values across domains.

Composite score two was constructed using **Consistent Intervals**, maintaining consistent scaling between the measurement of intervention domains and the values. The composite score is the sum of the values across the three domains, each ranging 0 to 1 (Table 1).

Composite score three employed a **Proportional Approach**, measuring engagement in the three intervention domains as a proportion of achieved U.S. POINTER prescription goals. The composite score is the sum of values across domains.

Composite score four utilized **Exploratory Factor Analysis** to identify optimal weighting for each domain’s adherence scores, aiming for a comprehensive assessment of participants' behavior.

Missing data are examined in two ways: (a) assume missing data indicates no adherence and assign zero, and (b) multiple imputation to predict missing values before composite calculation. Properties of the four composites are examined using simulated datasets in preparation for later use in U.S. POINTER. Distributions, central tendency, and variability of adherence values for each composite score will be presented.

**Conclusion:**

Four possible multidomain adherence composite scores are proposed, reflecting intervention engagement in U.S. POINTER. In the future, we will explore sensitivity of each composite score to detect treatment‐related change in cognition. This study will lay the foundation for broader applications in other multidomain trials with quantifiable adherence metrics, such as FINGER and LatAm‐FINGERS.